# Diagnostic value of harmonic transthoracic echocardiography in native valve infective endocarditis: comparison with transesophageal echocardiography

**DOI:** 10.1186/1476-7120-5-20

**Published:** 2007-05-19

**Authors:** Davinder S Jassal, Amin Aminbakhsh, Tielan Fang, Nasir Shaikh, John M Embil, Gordon S Mackenzie, James W Tam

**Affiliations:** 1 Bergen Cardiac Care Centre, Cardiology Division, Department of Cardiac Sciences, St. Boniface General Hospital, Winnipeg, Manitoba, Canada; 2 Institute of Cardiovascular Sciences, St. Boniface Research Centre, Winnipeg, Manitoba, Canada; 3 Section of Infectious Disease, Department of Medicine, Health Sciences Centre, Winnipeg, Manitoba, Canada; 4 Cardiovascular Anesthesia Division, Department of Cardiac Sciences, St. Boniface General Hospital, Winnipeg, Manitoba, Canada

## Abstract

**Background:**

Although echocardiography has been incorporated into the diagnostic algorithm of patients with suspected infective endocarditis (IE), systematic usage in clinical practice remains ill defined. To determine the diagnostic accuracy of detecting vegetations using harmonic transthoracic echocardiography (hTTE) as compared to transesophageal echocardiography (TEE) in patients with an intermediate likelihood of native valve IE.

**Methods:**

Between 2004 and 2005, 36 consecutive inpatients with an intermediate likelihood of disease were prospectively evaluated by hTTE and TEE.

**Results:**

Of 36 patients (21 males with a mean age of 57 ± 15 years, range 32 to 86 years), 19 patients had definite IE by TEE. The sensitivity for the detection of vegetations by hTTE was 84%, specificity of 88%, positive predictive value (PPV) of 89% and negative predictive value (NPV) of 82%. The association between hTTE and TTE interpretation for the presence and absence of vegetations were high (kappa = 0.90 and 0.85 respectively).

**Conclusion:**

In patients with an intermediate likelihood of native valve IE, TTE with harmonic imaging provides diagnostic quality images in the majority of cases, has excellent concordance with TEE and should be recommended as the first line test.

## Background

Infective endocarditis (IE) is a diagnostic and therapeutic challenge that is associated with high patient morbidity and mortality. [1] The diagnosis and management of IE have changed dramatically over the past 40 years, in particular the complementary use of echocardiography. [2,3] In addition to positive blood cultures and a new regurgitant murmur, echocardiographic findings has become one of the major Duke criteria for IE providing objective evidence of endocardial involvment. [3] Despite the higher sensitivity and specifity of transesophageal echocardiography (TEE) in the detection of valvular vegetations and characterization of complications, [4-7] transthoracic echocardiography (TTE) remains the initial procedure of choice in patients with suspected IE, due to its noninvasive nature and low cost. [8]

Although echocardiography has been incorporated into the diagnostic approach for patients with suspected IE, systematic usage in clinical practice is still not optimally defined. In patients with a high clinical likelihood of IE, the practical role of TTE for diagnostic purposes is low. [9,10] In the same context, echocardiography is often requested for patients with a transient fever, a nonregurgitant murmur, or both, who have a very low likelihood for the disease, with a low diagnostic yield. [9,10] Strict adherence to indications for TTE and TEE may help to facilitate more appropriate use and accurate diagnosis in patients who are most likely to benefit from screening echocardiography, in those patients with intermediate likelihood of the disease. [10]

Echocardiography using harmonic imaging (HI) is based on the principle of receiving double the emitted ultrasound frequency (fundamental and harmonic signals), separating out the two components, and processing the harmonic signal alone. [11] The properties of these harmonic signals are such that one can obtain significant improvements in spatial and contrast resolution, in particular of valvular structures, such as in the evaluation of suspected IE. [11] We thus sought to determine the diagnostic accuracy of detecting vegetations using harmonic transthoracic echocardiography (hTTE) as compared to TEE in patients with a intermediate likelihood of native valve IE at a Canadian tertiary care university hospital.

## Methods

### Patient population

Between 2004 and 2005, 98 consecutive adult inpatients referred to the University of Manitoba Health Sciences Centre Echocardiography Laboratory for the primary indication of "evaluation of suspected native valve endocarditis" were prospectively evaluated. Of the total population, 36 patients were classified as having intermediate likelihood of IE according to the adapted Duke criteria (Table 1). All 36 patients gave written informed consent to participate in the study, which was approved by the Biomedical Research Ethics Board of the University of Manitoba. The medical records of all 36 patients were extensively reviewed for baseline demographic data.

**Table 1:** Integrating clinical and laboratory data for rational use of echocardiography in patients with suspected native valve infective endocarditis

**Clinical criteria for diagnosis of infective endocarditis (adapting Duke Criteria)**
**Major criteria**
1. Positive blood cultures for infective endocaritis:
a. Typical microorganisms for infective endocarditis, including viridans strep, S. bovis, HACEK or community acquired Staph aureus or enterococcus OR
b. Microorganisms from persistent positive blood cultures, at least two positive cultures drawn >12 hours apart
2. Evidence of endocardial involvement:
a. New valvular regurgitation on clinical exam (worsening or changing of pre-existing murmur not sufficient)
**Minor criteria**
1. Predisposition: predisposing heart condition or intravenous drug use
2. Fever: temperature >38 C on two separate occasions
3. Vascular phenomenon: major arterial emboli, septic pulmonary infarcts, mycotic aneurysms, intracranial hemorrhage, conjunctival hemorrhages, Janeway lesions
4. Immunological phenomenon: glomerulonephritis, Osler's nodes, Roth's spots and rheumatoid arthritis
5. Microbiological evidence: positive blood cultures but does not a meet a major criteria as defined above
6. Serological evidence of active infection with organism consistent with endocarditis
**High Likelihood**: two major or one major and three minor clinical criteria
-Transthoracic and transesophageal echocardiography to assess prognosis or complications
**Intermediate likelihood**: one major or three minor clinical criteria
-TTE as initial test. If the echo is positive, then treat appropriately.
-TEE if the patient has high risk echocardiographic features on TTE or if clinical suspicion remains after negative or nondiagnostic TTE
**Low likelihood**: firm alternative diagnosis
-No echocardiography for diagnosis. Look for and treat alternative diagnosis

Reproduced with permission from Jassal DS et al. Can structured clinical assessment using modified Duke's criteria improve appropriate use of echocardiography in patients with suspected infective endocarditis? *Can J Cardiol *2003; 19 (9): 1017-22.

### Transthoracic and transesophageal echocardiography

All studies were performed with a Vivid 7, GE Medical System (Milwaukee, WI). Harmonic transthoracic echocardiography (hTTE) was performed first using a 1.5 MHz to 1.7 MHz transducer and TEE was performed within 24 hours of the hTTE, using a 4.5 MHz to 6.2 MHz multiplane transducer in all patients. Evidence of vegetations on echocardiography was predefined as a mobile or oscillating mass attached to the upstream endocardial surface of a native valve. [3]

The interpretation of all hTTE studies and the performance and interpretation of all TEE examinations were conducted by American Society of Echocardiography level III cardiologists as was routine within the local echocardiography laboratory. All images were graded as: i) positive; ii) indeterminate; or iii) negative for the presence of vegetations by two independent reviewers, blinded to the clinical findings. The reviewer of the TEE images was blinded to the hTTE results. In cases of nondiagnostic images or divergent results, the studies were reviewed by a third observer and consensus interpretation was achieved.

### Statistical analysis

The data are summarized as mean ± SD or number (percentage). Comparisons between the three image grades (positive, nondiagnostic, and negative) for hTTE and TEE respectively were made by Fisher's exact test or chi-square after Bonferroni correction. A p value < 0.05 was considered significant. The Statistical Analysis System 8.01 (SAS Insitute, Cary, NC) was used to perform the analysis.

## Results

### Baseline characteristics

**Figure 1:**
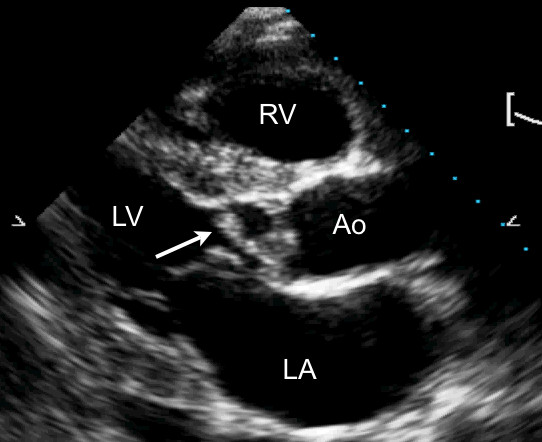
A transthoracic parasternal long axis view with harmonic imaging illustrating a vegetation (arrow) involving the aortic valve during diastole. LA, left atrium; LV, left ventricle; RV, right ventricle; Ao, aorta.

**Figure 2:**
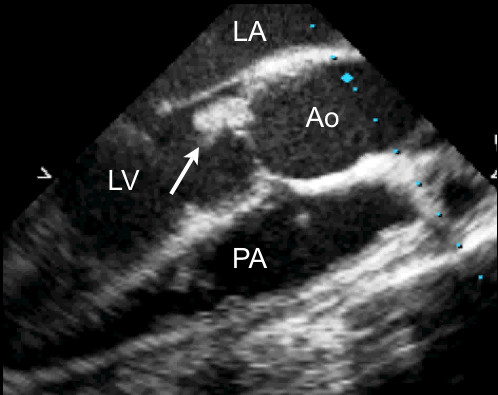
A transesophageal long axis view of the aorta in the same patient with demonstration of the vegetation on the noncoronary cusp of the aortic valve (arrow). LV, left ventricle; LA, left atrium; Ao; aorta; PA, pulmonary artery.

The total population included 36 patients with an intermediate likelihood of native valve IE (21 males with a mean age of 57 ± 15 years, range 32 to 86 years), who underwent both hTTE and TEE within 24 hours of each other. Baseline patient characteristics are listed in Table 2. The aortic valve was the most frequently affected in 13 (69%) patients (Figures 1, 2) [see additional files 1, 2] followed by the mitral valve in 6 (31%) patients. Of the discriminating factors listed in Table 1, 91% of patients fulfilled the DUKE criteria for typical blood cultures. The most commonly recovered pathogens were *Streptococcus viridans *followed by *Staphylococcus aureus*, accounting for 70% of the IE cases in this population. Amongst other clinical symptoms, 64% presented with fever, 28% presented with a vascular event, and approximately 25% had a higher propensity for valvular heart disease (degenerative valve disease, bicuspid aortic valve, or mitral valve prolapse).

**Table 2:** Baseline clinical characteristics of entire population (n = 36)

**Characteristics**	
Age	57 ± 15
Male gender (%)	21 (58)
Prior IE (%)	4 (11)
Rheumatic fever (%)	1 (3)
Fever (%)	23 (64)
Rheumatoid factor (%)	2 (6)
Valve disease (%)	9 (25)
Intravenous drug use (%)	3 (8)
Indwelling catheter (%)	2 (6)
New regurgitant murmur (%)	5 (14)
Positive blood cultures (%)	33 (91)
Vascular event (%)	10 (28)

Values are mean ± SD (percentage). y, years; IE, infective endocarditis.

### Detection of vegetations

The image quality for the diagnosis of vegetations identified by either hTTE or TEE are listed in Table 3. Of the total population, hTTE was diagnostic in 30 individuals (83%); positive in 16 patients and negative in 14 patients using TEE as the reference standard. The remaining 6 patients (17%) were indeterminate for the detection of vegetations by hTTE. All patients had diagnostic TEE for the presence or absence of vegetations, except for one individual who remained indeterminate due to complex valvular anatomy.

**Table 3:** Detection of vegetations by hTTE and TEE (n = 36)

Presence of vegetations	hTTE	TEE	p value
Positive	16	19*	0.46
Indeterminate	6	1*	<0.01
Negative	14	16	0.47

hTTE, harmonic transthoracic echocardiography; TEE, transesophageal echocardiography.

The sensitivity for the detection of vegetations by hTTE was 84%, specificity of 88%, positive predictive value (PPV) of 89% and negative predictive value (NPV) of 82% (Table 4). The association between hTTE and TEE interpretation for the presence and absence of vegetations were high (kappa = 0.90 and 0.85 respectively). Only 17% of patients with an intermediate likelihood of IE remained nondiagnostic after hTTE imaging, necessitating evaluation by TEE.

**Table 4:** Concordance rate for detection of vegetations by hTTE and TEE (n = 36)

Presence of vegetations	Positive TEE	Indeterminate TEE	Negative TEE
Positive hTTE	16	0	0
Indeterminate hTTE	3	1	2
Negative hTTE	0	0	14

hTTE, harmonic transthoracic echocardiography; TEE, transesophageal echocardiography.

## Discussion

Echocardiography is the primary technique for the detection of vegetations and cardiac complications resulting from IE. [2] Echocardiography provides one of the major Duke criteria including: (1) presence of vegetations defined as mobile echo-dense masses implanted in a valve or mural endocardium; (2) presence of abscess; or (3) presence of a new dehiscence of a valvular prosthesis. [3,13] The observation from this study is that in patients with an intermediate likelihood of native valve IE, TTE with harmonic imaging provides diagnostic quality images for detecting the presence or absence of vegetations in the majority of cases. Harmonic transthoracic echocardiography has excellent concordance with TEE imaging, and should be recommended as the first line test in this select patient population. If the clinical suspicion remains intermediate to high, however, or the hTTE remains equivocal due to structural abnormalities or poor acoustic windows, TEE imaging should be pursued.

Harmonic imaging has been in clinical use for over half a decade, emerging as a promising additional modality in echocardiography. [11,12] Harmonic imaging are disturbances of lower amplitude and higher frequency than the original vibration and when isolated, have frequencies of exact multiples of the fundamental frequency. [12] hTTE makes use of the second harmonic (twice the fundamental frequency) to minimize artefactual echoes that occur from indistinct tissue-tissue and tissue-blood interfaces. [12] As such, hTTE creates a cleaner echo signal with increased signal to noise ratio and improvement in lateral resolution (20–50%), at the cost of a reduction in axial resolution (40–100%). [14]

Transthoracic echocardiography using harmonic imaging has been extensively used in the clinical setting to enhance endocardial visualization, mitral valve assessment, and reproducibility of left ventricular systolic function, [15-17] but little is known about its use in the setting of suspected IE. In patients with a high pretest likelihood of IE (table 1), the practical role of TTE for diagnostic purposes is low, yet for prognostic purposes, it is useful for identifying perivalvular complications and assessing the severity of regurgitation. [9,10] Similarly, in patients with a low likelihood of disease who present with a transient fever and or nonregurgitant murmur, TTE has a low diagnostic yield, and an alternative diagnosis should be sought. [9,10]

The current study supports the use of hTTE in the setting where patients have an intermediate likelihood of suspected IE. If the image quality is interpretable using hTTE (>80% of our population), the concordance rate using TEE as the gold standard is high. These patients should be initially screened with hTTE to confirm the diagnosis, and if positive, treated appropriately. When the images are of good quality on hTTE and the study is negative, one should entertain an alternate diagnosis. TEE should be reserved for those patients with an indeterminate hTTE or in positive studies with high risk features.

The impact of hTTE has been recently evaluated for the detection of vegetations as compared to fundamental TTE and TEE, in a population predominantly of intermediate to high likelihood of IE. [18] Their overall sensitivity for identifying vegetations using hTTE of 82%, specificity of 98%, NPV of 95% and PPV of 93% was slightly higher as compared to our study. [18] As the pretest likelihood of IE was higher in their population, a stronger concordant rate between hTTE and TEE of 0.95 (χ^2 ^= 126, df = 4, r = 0.85 with p < 0.001) was detected. [18] Our population is more representative however of those patients in the intermediate pretest category of IE, who are most likely to benefit from screening echocardiography. In addition it was demonstrated that hTTE had greater sensitivity for the detection of vegetations on prosthetic valves, when compared to fundamental imaging. [18] Our study evaluated only those individuals with suspected native valve IE, as TEE should be reserved as the primary diagnostic imaging modality in patients with suspected prosthetic valve endocarditis. We did not, however, compare fundamental imaging using TTE in to hTTE in our population as experienced observers can readily distinguish the two forms of imaging leading to unblinding and potential bias. In comparison to TEE as the gold standard thus, hTTE was able to detect the presence of vegetations with high diagnostic accuracy in a population of intermediate likelihood of IE.

### Limitations

The primary limitation of this study is the small sample size, single-centre, and limited focus not addressing the other pretest categories of IE. Even though this study population of patients with intermediate likelihood of IE represents one of the largest of its kind in the diagnostic use of hTTE for IE, it remains small enough to be interpreted with caution. Although harmonic imaging is a major advance in echocardiography, the routine use of this technique in patients with suspected IE may cause false-positive findings as the valves appear thicker as compared to fundamental imaging. The absence of a group undergoing fundamental imaging in our study population is a limitation. A large prospective series or a multicentered approach may enable us to make more substantive conclusions regarding the role of hTTE in the diagnosis of patients with suspected IE.

## Conclusion

Echocardiography is an integral diagnostic modality in patients with suspected IE. The choice of initial mode of echocardiography is dependent on multiple factors including the patient's risk, native versus prosthetic valve and the pretest likelihood of infection. [19] Our study adds to the growing body of data supporting the role of initial TTE with harmonic imaging in patients with an intermediate likelihood of IE.

## Supplementary Material

Additional file 1 Aortic valve vegetation using harmonic TTE. This movie demonstrates an echodense mass attached to the aortic valve leaflet consistent with a vegetation using harmonic TTE imaging. Click here for file

Additional file 2 Aortic valve vegetation using TEE. This movie demonstrates an echodense mass attached to the noncoronary cusp of the aortic valve consistent with a vegetation using TEE imaging. Click here for file
